# Preliminary Study on the Sequencing of Whole Genomic Methylation and Transcriptome-Related Genes in Thyroid Carcinoma

**DOI:** 10.3390/cancers14051163

**Published:** 2022-02-24

**Authors:** Muhammad Asad Iqbal, Mingyang Li, Jiang Lin, Guoliang Zhang, Miao Chen, Nida Fatima Moazzam, Wei Qian

**Affiliations:** 1 Department of Otolaryngology-Head & Neck Surgery, Affiliated People’s Hospital of Jiangsu University, Zhenjiang 212002, China; 5103170106@stmail.ujs.edu.cn; 2 Department of Basic Medical Sciences, Affiliated to School of Medicine, Jiangsu University, Zhenjiang 212002, China; limy@ujs.edu.cn; 3 Laboratory Center, Affiliated People’s Hospital of Jiangsu University, Zhenjiang 212132, China; linjiangmail@sina.com; 4 Department of General Surgery, Affiliated People’s Hospital of Jiangsu University, Zhenjiang 212132, China; zhgl200208@163.com; 5 Department of Pathology, Affiliated People’s Hospital of Jiangsu University, Zhenjiang 212132, China; 18912802271@163.com; 6 School of Medicine, Jiangsu University, Zhenjiang 212013, China; 5103160101@stmail.ujs.edu.cn

**Keywords:** thyroid carcinoma, targeted bisulfite sequencing assay, DNA methylation, MethylationEPIC BeadChip (850K), RNA-Seq

## Abstract

**Simple Summary:**

Epigenetic alterations are critical for tumor onset and development. DNA methylation is one of the most studied pathways concerning various types of cancer. A promising and exciting avenue of research is the discovery of biomarkers of early-stage malignancies for disease prevention and prognostic indicators following cancer treatment by examining the DNA methylation modification of relevant genes implicated in cancer development. We have made significant advances in the study of DNA methylation and thyroid cancer. This study is novel in that it distinguished methylation changes that occurred primarily in the gene body region of the aforementioned hypermethylated or hypomethylated thyroid cancer genes. Our findings imply that exposing whole-genome DNA methylation patterns and gene expression profiles in thyroid cancer provides new insight into the carcinogenesis of thyroid cancer, demonstrating that gene expression mediated by DNA methylation modifications may play a significant role in tumor growth.

**Abstract:**

Thyroid carcinoma is the most prevalent endocrine cancer globally and the primary cause of cancer-related mortality. Epigenetic modifications are progressively being linked to metastasis. This study aimed to examine whole-genome DNA methylation patterns and the gene expression profiles in thyroid cancer tissue samples using a MethylationEPIC BeadChip (850K), RNA sequencing, and a targeted bisulfite sequencing assay. The results of the Illumina Infinium human methylation kit (850K) analyses identified differentially methylated CpG locations (DMPs) and differentially methylated CpG regions (DMRs) encompassing nearly the entire genome with high resolution and depth. Gene ontology and KEGG pathway analyses revealed that the genes associated with DMRs belonged to various domain-specific ontologies, including cell adhesion, molecule binding, and proliferation. The RNA-Seq study found 1627 differentially expressed genes, 1174 of which that were up-regulated and 453 of which that were down-regulated. The targeted bisulfite sequencing assay revealed that CHST2, DPP4, DUSP6, ITGA2, SLC1A5, TIAM1, TNIK, and ABTB2 methylation levels were dramatically lowered in thyroid cancer patients when compared to the controls, but GALNTL6, HTR7, SPOCD1, and GRM5 methylation levels were significantly raised. Our study revealed that the whole-genome DNA methylation patterns and gene expression profiles in thyroid cancer shed new light on the tumorigenesis of thyroid cancer.

## 1. Introduction

Thyroid carcinoma is currently considered to be induced by the multi-step process of carcinogenesis, in which cancer cells are formed from thyroid follicular cells (thyroid epithelial cells) via numerous incidences of genomic injury. These injuries primarily occur in oncogenes and anti-oncogenes that promote proliferation or the development of malignant phenotypes, such as the ability to penetrate surrounding tissue or metastasize to distant organs [1,2]. Thyroid carcinomas are a frequently occurring type of endocrine cancer that exhibits varying phenotypes, ranging from mild forms to the most aggressive forms of human cancer. Thyroid carcinomas are classified into several types, including well-differentiated thyroid carcinoma (WDTC), undifferentiated thyroid carcinoma (UTC), poorly differentiated thyroid carcinoma (PDTC), anaplastic thyroid carcinoma (ATC), and medullary thyroid cancer (MTC). Of these, WDTC, UTC, PDTC, and ATC are all derived from thyrocytes; in contrast, MTC is derived from C cells. Furthermore, differentiated thyroid cancer is divided into three primary subtypes: papillary thyroid carcinoma (PTC), follicular thyroid carcinoma (FTC), and Hürthle cell cancer. Differentiated thyroid carcinomas account for ninety-five percent of all thyroid cancers globally. Numerous epidemiological studies have found that the incidence of differentiated thyroid cancer has increased significantly over the last few centuries [3]. The vast majority of thyroid carcinomas present as thyroid nodules, which are detected by the physician during a physical examination or during neck imaging for other disorders. Thyroid nodules can be cancerous in a small percentage of cases. Thyroid nodules identified in the general population have a 5–10% chance of being cancerous, although men and patients at the extremes of age are at a greater risk [4]. A considerable proportion of patients with well-differentiated thyroid carcinoma are treated with a total thyroidectomy, including the excision of the anterior or central compartment lymph nodes, radioactive iodine therapy for abscission of metastases and thyroid remnants, and suppression of TSH with l-thyroxin [5].

Deeper knowledge of the molecular pathways underlying the growth of thyroid cancer might be critical for tailoring treatments. Over the last three decades, significant progress has been made in this area [6]. There are several epigenetic mechanisms: DNA methylation, chromatin remodeling, and post-translational histone modifications. These mechanisms have been studied elsewhere [7,8,9,10,11]. DNA methylation is a long-lasting epigenetic modification that has identified in cancer for over three decades [12]. As a gene silencing mechanism, DNA methylation is necessary for the proper development and operation of several structures and cellular processes, including embryogenesis, transcription, X-inactivation, and genomic imprinting [13,14,15,16]. In humans, DNA methylation occurs nearly entirely within CpG dinucleotides, which are underrepresented, i.e., they occur less frequently than estimated based on the GC composition of DNA and are not evenly distributed throughout the genome [17]. The vast majority of the human genome is methylated, with approximately 60–80 percent of CG sites methylated, with the exception of selected CpG-rich sections, known as CpG islands or CG islands (CGIs), that are commonly unmethylated and contain the promoters for approximately 60 percent of all protein-coding genes [18,19,20].

Although prior studies have shed light on the association between gene regulation and DNA methylation in the development of thyroid cancer, the overall knowledge base remains extremely limited. Multiple studies have demonstrated that aberrant DNA methylation patterns in cancerous tissues may mute tumor suppressor genes while activating oncogenes via hypermethylation/hypomethylation [21,22]. However, hypermethylation is more frequently reported than hypomethylation in cancers [23]. Some genes, including *MLH1* and *p16INK4A*, are frequently hypermethylated in various malignancies, including thyroid carcinoma. The tumor-specific sodium iodide symporter gene, *NIS* (also known as *SLC5A5*), is also expressed [24,25,26,27]. Across all of the different epigenetic alterations, DNA methylation on CpG islands is the most widely researched. It is well known that hypermethylation of CpG islands in a gene’s promoter region suppresses its expression. Furthermore, changes in DNA methylation have been observed to arise in the initial stages of oncogenesis, implying that they could be exploited as a viable biomarker for cancer detection [28,29,30]. Several DNA methylation-based biomarkers have been reported in various cancers, including stomach cancer, prostate cancer, bronchial carcinoma, and bowel cancer [31,32,33,34]. The initial studies on the effects of DNA methylation in thyroid carcinoma were performed using a candidate gene approach that assessed the DNA methylation level of particular gene promoters [35].

In this study, our primary goal is to detect local differentially methylated CpG regions (DMRs) between thyroid cancer and normal thyroid tissue groups at a genome-wide level. We found 43,653 significantly differentially methylated CpG positions (DMP), accounting for 6.10% of all possible DMPs, and 236 significantly differentially methylated CpG regions, accounting for 18.96% of all possible differentially methylated regions (Supplementary Figure S1). Gene Ontology and KEGG enrichment analysis of differentially methylated and differentially expressed genes (DEGs) revealed that the genes involved in DNA methylation were significantly enriched in the regulation of the phosphatidylinositol 3-kinase/protein kinase B (PI3K-Akt), human papillomavirus (HPV) infection, and mitogen-activated protein kinase (MAPK) signaling pathways. This indicates that methylation-related genes are highly enriched in malignancy-related pathways. In this investigation, we identified 1627 genes expressed differently in tumor tissue than in the adjacent healthy tissue (Supplementary Table S2). In comparison to the nearby normal tissue, the tumor tissue had 453 genes with downregulated expression and 1174 genes with elevated expression. It is yet to be determined which specific differentially methylated genes (DMG) are implicated in thyroid cancer. The hypermethylated and low-expressed genes (thyroid tumor vs. normal control) were intersected, yielding seven genes. The hypomethylated and high-expressing genes (thyroid tumor vs. normal control) were intersected, yielding 65 genes (Supplementary Table S3). The validation of thyroid tumor-related genes reveals that the methylation levels of *CHST2*, *DPP4*, *DUSP6*, *ITGA2*, *SLC1A5*, *TIAM1*, *TNIK*, and *ABTB2* were significantly lower in thyroid cancer patients when compared to the controls, while the methylation levels of GALNTL6, HTR7, SPOCD1, CDH16 and GRM5 were significantly higher (Supplementary Table S5, Figure S8). The thyroid tumor-related genes have been validated. Differently methylated genes are identified using targeted bisulfite sequencing, and differentially expressed genes are identified using RQ-PCR.

## 2. Material and Methods

### 2.1. Tissue Samples of Patients

**Figure 1:**
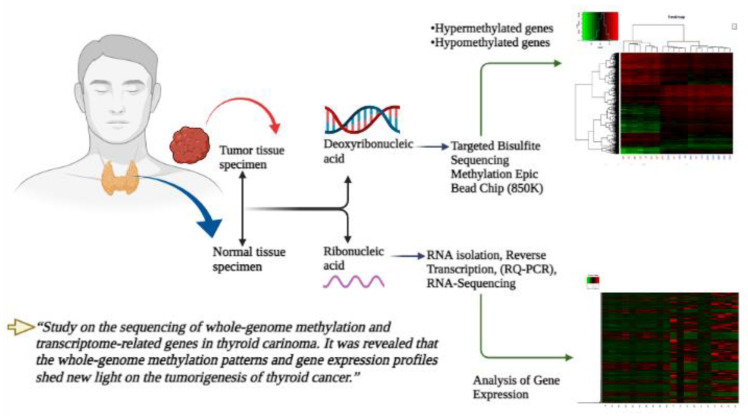
The overall structure and the methods used for methylation analysis in the thyroid cancer samples.

Pairs of fresh frozen thyroid carcinoma samples and adjacent normal thyroid carcinoma tissue samples were obtained. Initially, we acquired ten pairs of malignant thyroid cancer tissues and normal thyroid tissues, then we went through a quality check of the specimens. We collected a total of 86 matched samples of pathologically verified post-operative malignant carcinoma and normal thyroid tissues from 43 specimens, from patients who underwent thyroidectomy at the Zhenjiang First People’s Hospital, affiliated with the Institute of Jiangsu University, between July 2018 and September 2020. The overall structure and the methods used in this study are shown in Figure 1. All procedures were conducted following the appropriate norms of Affiliated People’s Hospital of Jiangsu University’s Ethics Board. After surgery, the eighty-six specimens were immediately frozen in liquid nitrogen and preserved at −80 °C. Neither preoperative chemotherapy nor radiotherapy had been administered to the patients selected for this study, and the specimens contained cancerous tissue. The controls were normal thyroid tissues more than 2 cm away from the tumor and did not have infiltrated cancer cells. This study was approved by the Ethics Committee of the Affiliated People’s Hospital of Jiangsu University, and written informed consent was taken from all participants prior to their inclusion.

### 2.2. MethylationEPIC BeadChip (850K)

It was determined that the replication cohort’s DNA was methylated using the Infinium MethylationEPIC BeadChip (850K) (Illumina, Inc., San Diego, CA, USA). The R Package ChAMP was used to analyze, normalize, and perform differential methylation analysis on the genome-wide methylation data. Genomic DNA was extracted from cells using the NucleoSpin Tissue kit (Macherery-Nagel, GmbH & Co. KG, Düren, Germany). DNA (cytosine) methylation profiles were generated utilizing an array, by combining bisulfite conversion and whole-genome results amplified with the direct captures and scores of CpG (cytosine-guanine) loci. DNA specimens were processed and hybridized to the human Infinium MethylationEPIC BeadChip (Illumina, San Diego, CA, USA), designed to quantitatively assay over 850K methylation sites across the genome using the Infinium HD Methylation Assay protocol. Hybridized BeadChips were scanned according to the manufacturer’s specifications using an Illumina iScan system. Annotation of the genes was carried out utilizing the annotation provided with Illumina’s probe. To summarize, CpG markers were categorized on the MethylationEPIC 850K array according to their chromosomal position. Marker Infinium (I), Infinium II, and the UCSC annotation feature’s gene area category were studied using Infinium Chemistry [36]. To make the datasets more interpretable, principal component analysis (PCA) reduced their dimensionality while avoiding information loss. This is accomplished by successively increasing the variance of uncorrelated variables. Hierarchical clustering analysis is a comparable technique for grouping similar objects into clusters. The endpoint is a collection of clusters, each distinct from the others, but containing broadly similar objects. 

### 2.3. RNA Sequencing (RNA-Seq)

Genome-wide gene expression analysis of thyroid carcinoma was performed utilizing second-generation RNA sequencing (RNA-Seq). A commercial company evaluated the RNA-Seq data (BGI, Beijing, China). The log2 ratio of the unfiltered air data to the filtered air data represents the gene expression results. Total RNA was extracted according to the manufacturer’s instructions using a combination of TRIzol™ Reagent and ethanol for precipitation (Tiangen Biotech, Beijing, China). A spectrophotometer and an Agilent 2100 Bioanalyzer were used to evaluate the samples’ integrity and RNA content (Agilent Technologies, Inc., Santa Clara, CA, USA).

### 2.4. Targeted Bisulfite Sequencing Assay

MethylTarget (Genesky, Shanghai, China) was used to perform targeted bisulfite sequencing. MethylTarget is a next-generation sequencing (NGS) platform. As previously mentioned, DNA extraction and bisulfite conversion were carried out [37,38]. To identify the different probable CpG sites in a panel of samples, we meticulously constructed primers based on the genome coordinates of the regions. A net polymerase chain reaction was utilized to amplify the desired DNA sequence, which was then purified. An Illumina HiSeq 2000 sequencing system was then used to sequence the intended DNA fragments. BS-Seeker2, a widely used tool for evaluating bisulfite sequencing data, was employed in our investigation to map bisulfite-treated reads and detect methylation [39]. To complete our methylation process, we examined the bisulfite conversion rates that corresponded to each sample. We then used only samples with a bisulfite conversion rate of 98 percent. After performing a preliminary analysis, we found that each CpG site receives an average of 96% of the nucleotides, and that the missing rate is about 4%. Following additional filtering, sites with coverage less than 20 and sites with a missing rate higher than 0.20 were eliminated. When more than 30% of the samples tested were missing, those samples were also removed.

### 2.5. RNA Isolation, Reverse Transcription and Real-Time Quantitative PCR (RQ-PCR)

As previously shown, total RNA isolation and reverse transcription were carried out [40]. RQ-PCR was used to validate the significantly up- or down-regulated genes as identified by RNA-Seq analysis. Real-time quantitative PCR (RQ-PCR) was used to detect the expression of the target/screened genes, as previously explained [41]. The study was performed using an BioRad Opticon 2 qPCR machine (Hercules, CA, USA) using an SYBR Green mix (Tiangen Biotechnology, Beijing, China) and the following cycling program: ten min at 95 °C, forty cycles at 95 °C for 25 s, and one min at 60 °C. The internal control for normalization was glyceraldehyde 3-phosphate dehydrogenase (GAPDH) and every sample was ran three times. A set of primers was used to amplify each cDNA aliquot [42].

### 2.6. Analytical Statistics

SPSS version 27.0.1 and Prism GraphPad 9.2.0 were utilized for statistical analysis. The Wilcoxon rank-sum test was used to contrast two groups of continuous variables, while Pearson’s chi-squared test was used to differentiate two groups of categorical data. The correlation analysis was conducted using Spearman’s rank correlation coefficient. The statistically significant value was set at *p* < 0.05 for all tests and conducted on a two-sided basis [42]. The analysis of Gene Ontology (GO) and KEGG (Kyoto Encyclopedia of Genes and Genomes) enrichment was carried out using the R package clusterProfiler. 

## 3. Results

### 3.1. Analysis of Genome-Wide Methylation in the Thyroid Carcinoma Patients

**Figure 2:**
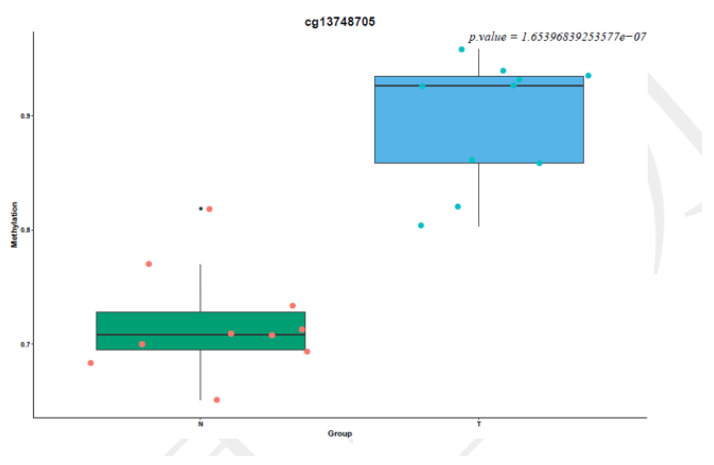
Box plots comparing the degree of methylation of grouped samples for the top 300 most significantly differentially methylated CpG positions. The title indicates the name of the probe, the *X*-axis indicates the grouping information, the *Y*-axis indicates the degree of methylation of each grouped sample, and in the upper right corner is the *p*-value of the probe, as determined by further analysis. The scatter plot shows each sample’s specific methylation degree value, and the box plot shows the difference in the methylation degree distribution between the two groups. The green dots represent thyroid carcinoma from patients, while the red dots represent their adjacent normal tissues.

**Figure 3:**
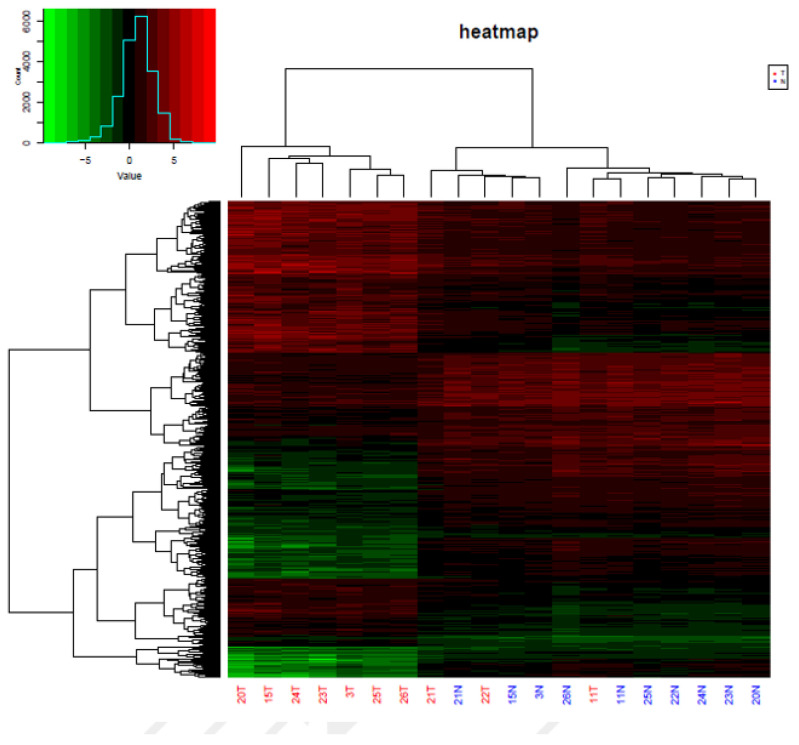
Heat map results of genes where differential methylation sites are located. Each column represents a sample, and each row represents a gene where a differentially methylated site is located. The value of the sample at a given site = log2 (degree of methylation/(degree of 1-methylation)).

To identify epigenetic alterations occurring in thyroid carcinoma patients, the genome-wide methylation patterns were explored using the Illumina Infinium Human Methylation Assay (850K) in a total of 10 newly diagnosed thyroid carcinoma patients’ tumors and their adjacent tissues. A mean of 864,187 probes per sample was qualified to detect CpG (*p*-value < 0.01) (Supplementary Table S1). We identified 43,653 significantly differentially methylated CpG positions (DMP), accounting for 6.10% of all possible DMPs, and 236 significantly differentially methylated CpG regions (DMR) accounting for 18.96% of all possible DMRs. The methylation density plot is shown in Supplementary Figure S1. The PCA and hierarchical cluster analysis (HCA) of normal controls and thyroid carcinoma patients based on CpG sites’ methylation differentiated the thyroid carcinoma patients from the controls (except for the three tumor patients labeled as 21T, 22T, and 11T, Supplementary Figures S2 and S3). For the top 300 most significantly differentially methylated CpG positions, tumors from the thyroid carcinoma patients showed drastically increased DNA methylation levels when compared with their normal tissues (Figure 2). Differentially methylated genes were clustered together into hypermethylated and hypomethylated genes, which is obviously displayed in the heat map comparing tumor patients and normal controls (Figure 3).

### 3.2. Functional Analysis of Genes Associated with DNA Methylation in Thyroid Cancer Patients

**Figure 4:**
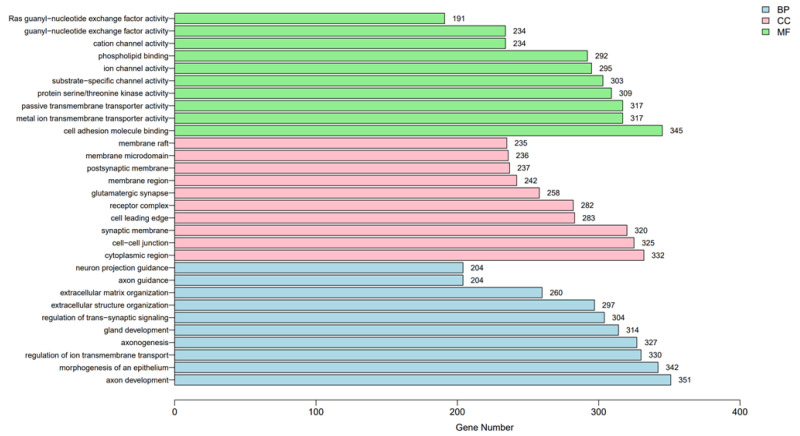
GO enrichment analysis results of genes with significant methylation differences.

**Figure 5:**
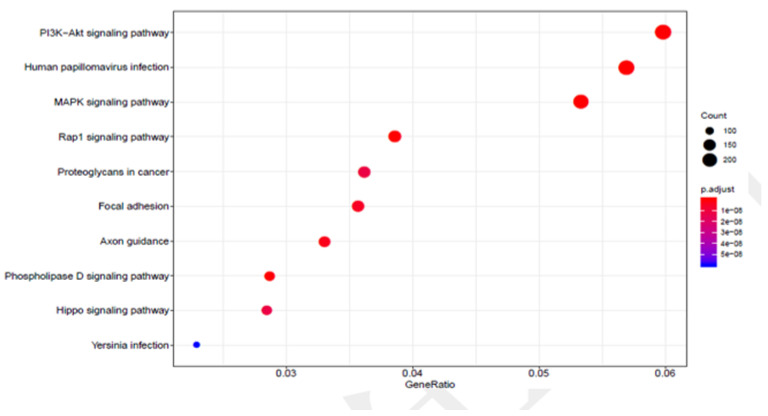
KEGG enrichment analysis results of genes with significant methylation differences.

**Figure 6:**
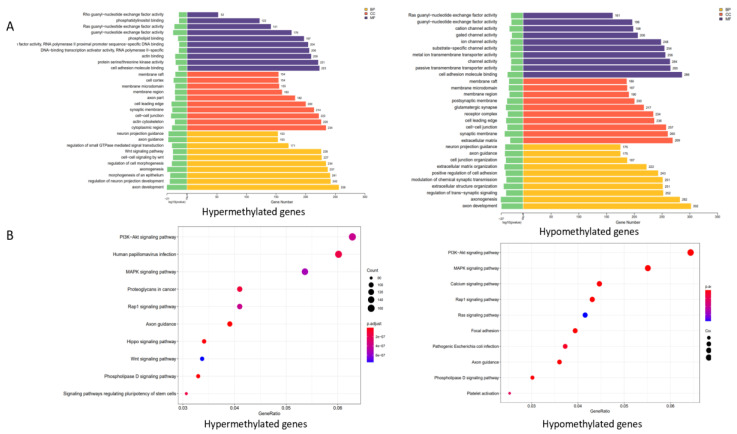
GO and KEGG enrichment analysis results of genes with significant methylation differences. (**A**) GO enrichment analysis of hypermethylated and hypomethylated genes. (**B**) KEGG enrichment analysis of hypermethylated and hypomethylated genes.

To better understand the potential functions of the DNA methylation-related genes, we conducted a Gene Ontology functional enrichment analysis and a Kyoto Encyclopedia of Genes and Genomes pathway enrichment analysis. For biological processes, the results suggested that genes involved in DNA methylation were considerably enriched in terms of cell communication; though material transport, including cell adhesion molecule binding; metal-ion passive transmembrane transporter activity; Ras guanyl-nucleotide exchange factor activity; and cation-ion/substrate-specific channel activity (Figure 4). The KEGG pathway enrichment analysis is shown in Figure 5. The results showed that the regulation of the phosphatidylinositol 3-kinase/protein kinase B (PI3K-Akt), human papillomavirus (HPV) infection, and mitogen-activated protein kinase (MAPK) signaling pathways were considerably enriched in DNA methylation genes. They also indicated that methylation-related genes are significantly enriched in malignancy-related pathways. A comparison of hypermethylated and hypomethylated genes using the KEGG enrichment analysis is given in (Figure 6).

### 3.3. The Alterations in Gene Expression Related to Thyroid Carcinoma

**Figure 7:**
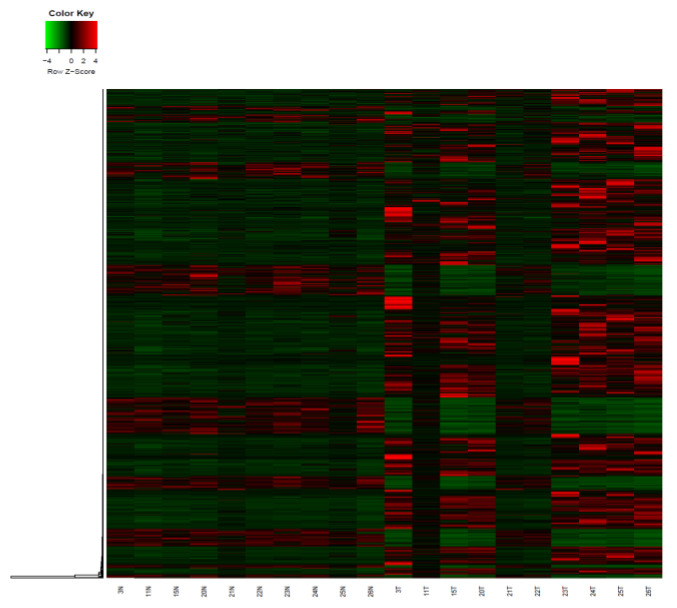
Heat map results of genes that are differentially expressed between patients and normal controls. Each column represents a sample, and each row represents a differentially expressed gene.

Transcriptome analysis of paired tumors and adjacent normal tissues from 10 patients used RNA-Seq was performed to identify changes in gene expression patterns in thyroid cancer. A total of 19,476 genes were investigated for gene expression through RNA-Seq in the tumor patients and normal controls, among which we identified 1627 genes that were expressed differently in tumor tissue, as compared to nearby healthy tissue in the study (Supplementary Table S2). In comparison to the nearby normal tissue, the tumor tissue had 453 genes with down-regulated expression and 1174 genes with elevated expression. Figure 7 shows a heat map of genes that were significantly up- or down-regulated between tumors and healthy tissues.

### 3.4. Screening of Candidate Differentially Methylated Genes Related to Thyroid Cancer

To identify the candidate DMGs involved in thyroid cancer, we screened genes by the following process: first, in the differential methylation results, *p*-value < 0.001 and |meth. diff| > 0.25 were thresholds to screen out differentially methylated genes. Then, in the differential expression results from whole transcriptome sequencing, *p*-value < 0.05 and |log2FoldChange| > 1.5 were used as thresholds to screen out differentially expressed genes. Finally, the hypermethylated and low-expressed genes (thyroid tumor vs. standard control) were intersected, and seven genes were obtained. The hypomethylated and high-expressing genes (thyroid tumor vs. standard control) were intersected, and 65 genes were obtained (Supplementary Table S3).

### 3.5. Identification and Validation of Thyroid Tumor-Related Genes by Targeted Bisulfite Sequencing and RQ-PCR

After excluding 17 unqualified genes due to them not containing CpG islands, not being feasibile to design primers for, or according to their expression profile, the other 55 DMGs were selected for further validation to give a robust characterization of the methylation state of the CpG sites. Bisulfite sequencing in an additional 36 patients with thyroid carcinoma and 41 normal controls was performed using MethylTarget (Genesky, Shanghai, China), which is based on a next-generation sequencing (NGS) platform. We analyzed 119 DNA target fragments (100–300 bp) from the 55 DMGs and assessed the CpG site-specific methylation levels within each DNA fragment using bisulfate sequencing (Supplementary Table S4). 

**Figure 8:**
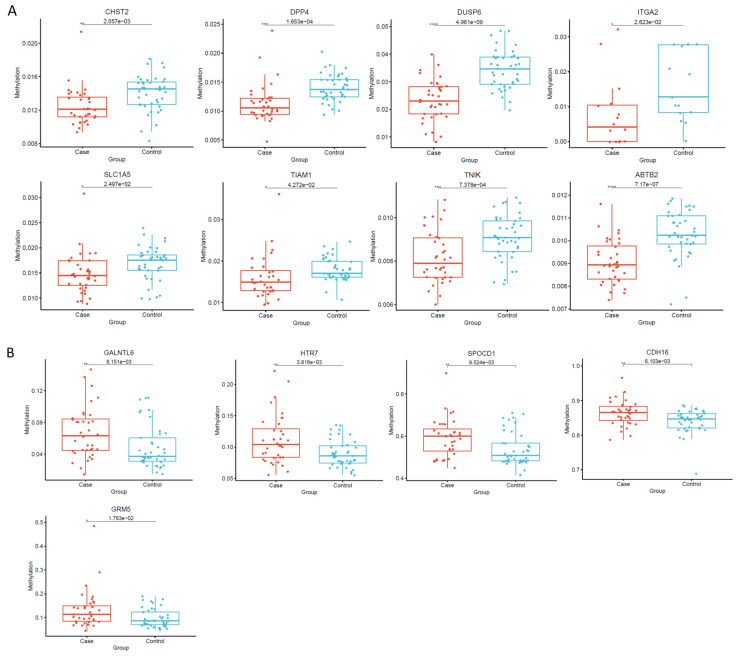
Box plot of the average methylation levels of interesting genes. The name of the image is the name of the target gene. The colors of the dots represent the different groups. The *X*-axis is the two groups for further analysis. The *Y*-axis is the average degree of methylation of each sample in the group on the target gene. The box represents the group. Each dot represents each sample’s average degree of methylation on the target gene. (**A**) CHST2, DPP4, DUSP6, ITGA2, SLC1A5, TIAM1, TNIK, and ABTB2 are hypomethylated in thyroid carcinoma patients when compared to normal control tissues. (**B**) GALNTL6, HTR7, SPOCD1, CDH16 and GRM5 are hypermethylated in thyroid carcinoma patients when compared to normal control tissues. *: *p* < 0.05, **: *p* < 0.01, ***: *p* < 0.001, ****: *p* < 0.0001.

The methylation levels of CHST2, DPP4, DUSP6, ITGA2, SLC1A5, TIAM1, TNIK, and ABTB2 were markedly decreased in thyroid cancer patients when compared with the controls, while the methylation levels of GALNTL6, HTR7, SPOCD1, CDH16 and GRM5 were significantly increased (Supplementary Table S5, Figure 8). In addition, the real-time quantitative PCR (RQ-PCR) results showed that all of the aforementioned genes presented significant differential expression between the 42 patients with thyroid tumors and the 43 healthy controls. All of these genes showed consistent regulation direction within the samples (Supplementary Table S6).

## 4. Discussion

Epigenetic modifications play a vital role in the initiation and progression of tumors. DNA methylation has become one of the most investigated mechanisms related to different cancers. It would be very great and promising to discover biomarkers of the early stages of tumors to prevent diseases and prognostic markers following the treatment of cancers by examining the DNA methylation alterations of exciting genes involved in cancer development. In recent years, many efforts have been made by the scientific community in the field of DNA methylation related to thyroid cancer [43,44,45,46]. There is a strong relationship between genetic alterations and epigenetic aberrations in tumorigenesis [47]. It is widely assumed that genetic changes, particularly alternate DNA methylation levels, can significantly impact abnormal gene expression of critical genes, leading to the development and progression of tumors by facilitating or inhibiting transcription factor binding for transcriptional activity [48]. We utilized techniques to focus on the genome-wide level of DNA methylation as to distinguish the hypermethylated and hypomethylated genes and to study the expression level of these genes on a series of paired tumor and adjacent normal tissue samples from patients who acquired thyroid carcinoma in the local region of our city in China. We then selected a few candidate genes which were hypermethylated or hypomethylated in tumor samples as compared to healthy tissues, and then executed a targeted bisulfite sequencing technique to investigate the methylation status of CpG sites in the interesting genes’ promoter regions, as well as their adjacent genomic regions, therefore producing a robust measure of the methylation level of the candidate genes.

Several hypermethylated genes (GALNTL6, HTR7, SPOCD1, CDH16 and GRM5) related to thyroid cancer have been discovered to be up-regulated in our study, as were investigated in other reports to mentioned in the following text. GRM5 is a metabotropic glutamate receptor gene that encodes a protein from the G-protein coupled receptor 3 protein family. Its signaling causes a phosphatidylinositol-calcium secondary messenger system to be activated. GRM5 was highly expressed in oral squamous cell carcinoma and contributed to tumor cell migration and invasion [49]. Inhibiting GRM5 expression could suppress oncogenic actions by blocking downstream signaling factors in hepatocellular carcinoma [50]. HTR7 also belongs to the G protein-coupled receptor (GPCR) family. One recent study demonstrated high expression in laryngeal cancer tissues promoted tumor proliferation by activating the PI3K/AKT pathway [51]. SPOCD1 was up-regulated and promoted cell proliferation in osteosarcoma cell lines [52]. It has also been reported that the significantly expressed SPOCD1 accelerated the progression of ovarian carcinoma and inhibited cell death (apoptosis) via the PI3K/AKT pathway [53]. There are currently no reports regarding the high expression of GALNTL6 in cancers. The role of the up-regulation of GALNTL6 in thyroid cancer is still unknown and is worthy of investigation. 

We have also found a few hypomethylated genes, CHST2, DPP4, DUSP6, ITGA2, SLC1A5, TIAM1, TNIK, and ABTB2, which showed high expression in thyroid carcinoma. Transfer of a sulfate residue to GlcNAc residues in keratan sulfate by CHST2 has been revealed to activate the p38 MAPK-PI3K (mitogen-activated protein kinase/phosphatidylinositol 3-kinase) cell signaling pathway and decrease cell apoptosis caused by radiation in Burkitt’s lymphoma cells [54]. Aberrantly high levels of DPP4 expression occurred in human hepatocellular carcinoma [55]. For the gene DUSP6, a previous study [56] showed that its gene expression increased in all of studied thyroid cancer cell lines, consistent with our results. It also reported that upregulation of DUSP6 gene transcription in human glioblastoma played a tumor-promoting role and accelerated the malignancy of tumors [57]. For ITGA2, SLC1A5, TIAM1, TNIK, and ABTB2, several studies showed that their high expression played a significant role in promoting tumor cell growth and inhibiting cell apoptosis from chemotherapy in different kinds of cancers [58,59,60,61,62]. The most intriguing discovery in our study is that almost all the significant differential methylation alterations occurred exclusively in the region of the gene body of the aforementioned hypermethylated/hypomethylated genes involved in thyroid carcinoma. Gene body methylation is positively correlated with gene expression levels, though the mechanisms are unclear [63]. One recent study revealed that the investigated genes possessed an exceptional hypermethylation level of the CpG islands located in the gene body region, and all of them were simultaneously overexpressed in hepatocellular carcinoma [64]. This phenomenon was believed to be predictive of increased oncogene levels in cancer. Further study needs to be performed to focus on the mechanism behind the methylation alterations in the gene body of these interesting genes in thyroid carcinoma. The results from our study could give potential opportunities to identify new drug targets in thyroid cancer [65].

## 5. Conclusions

In conclusion, our integrative analysis provides a new perspective that gene expression regulated by DNA methylation alterations located primarily in the gene body may play a crucial role in the progression of tumors and that DNA methylation levels of critical genes could be reverted to normal by methylation or demethylation drugs for the treatment of cancers. Furthermore, these discovered genes can be potentially used as biomarkers for predicting the development of thyroid cancer.

## Data Availability

No new data were created or analyzed in this study. Data sharing is not applicable to this article.

## Supplementary Materials

The following supporting information can be downloaded at: https://www.mdpi.com/article/10.3390/cancers14051163/s1, Figure S1: The density distribution plot of the methylation degree of each sample probe after normalization (Note: The *X*-axis is the degree of methylation, and the *Y*-axis is the probe density corresponding to the degree of methylation. Each curve represents a sample, and the color distinguishes the sample group); Figure S2: PCA analysis results of methylation ratio of each sample (Note: Color distinguishes sample groups; *X* and *Y* axes respectively correspond to the indicators that best reflect the true species composition of the sample, calculated by software. Number represents patients label number, and the following letter N represents patients’ normal control while letter T represents patients’ thyroid carcinoma); Figure S3: Hierarchical clustering results of methylation ratio of each sample. Number represents patients label number, and the following letter N represents patients’ normal control while letter T represents patients’ thyroid carcinoma. Table S1: The number of qualified probes of the sample and the average intensity of the red and green signals of all probes (Note: A maximum of 10 samples are displayed. “Sample Name” refers to the name of each sample; “Detected CpG (0.01)” refers to the number of qualified probes for the sample under the condition that the pvalue threshold is 0.01, that is, the pvalue of the retained probe in all samples is less than 0.01, “Detected CpG (0.05)” refers to the number of qualified probes for the sample under the condition that the pvalue threshold is 0.05, that is, the pvalue of the retained probes in all samples is less than 0.05. “Signal Average GRN” refers to all the samples in each sample. The average intensity of the green signal of the probe, “Signal Average RED” refers to the average intensity of the red signal of all probes for each sample); Table S2: The differential expression of genes between thyroid carcinoma patients and their normal control tissues; Table S3: The screening results of candidate differentially methylated genes related to thyroid cancer; Table S4: The differential methylation level of all target fragments of interested genes by targeted bisulfite sequencing in thyroid cancer; Table S5: The differential methylation level of interested genes by targeted bisulfite sequencing in thyroid cancer; Table S6: the quantitative RT-PCR results showing expression level of targeted genes involved in thyroid cancer.
