# Supplementary material for: Preliminary Study on the Sequencing of Whole Genomic Methylation and Transcriptome-Related Genes in Thyroid Carcinoma
Source: Cancers (Basel). 2022 Feb 24;14(5):1163. doi: 10.3390/cancers14051163 (PMC8909391; doi:10.3390/cancers14051163)
Supplement: Supplementary file 1 [file cancers-14-01163-s001.zip › Figures and Table S1.pdf]

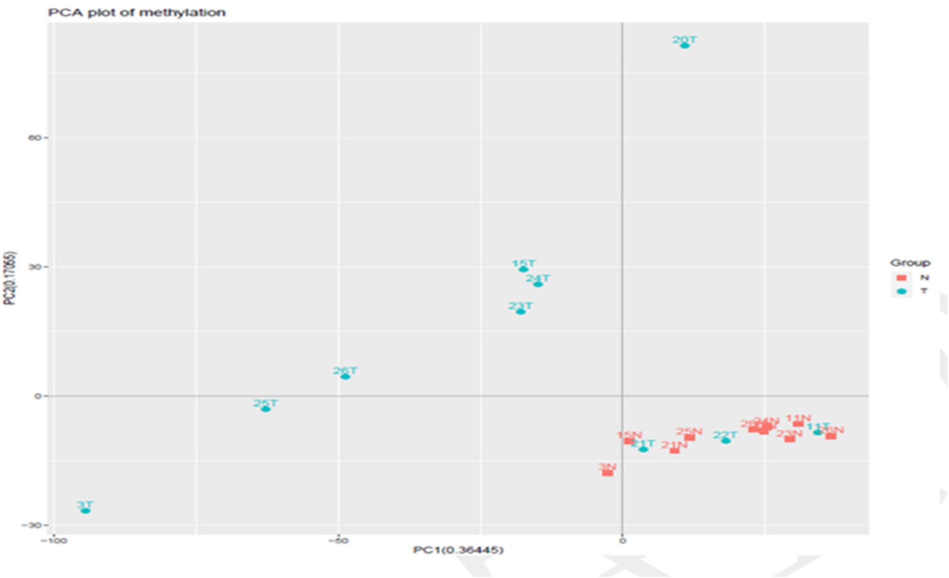

Figure S1. The density distribution plot of the methylation degree of each sample probe after normalization (Note: The X-axis is the degree of methylation, and the Y-axis is the probe density corresponding to the degree of methylation. Each curve represents a sample, and the color distinguishes the sample group).

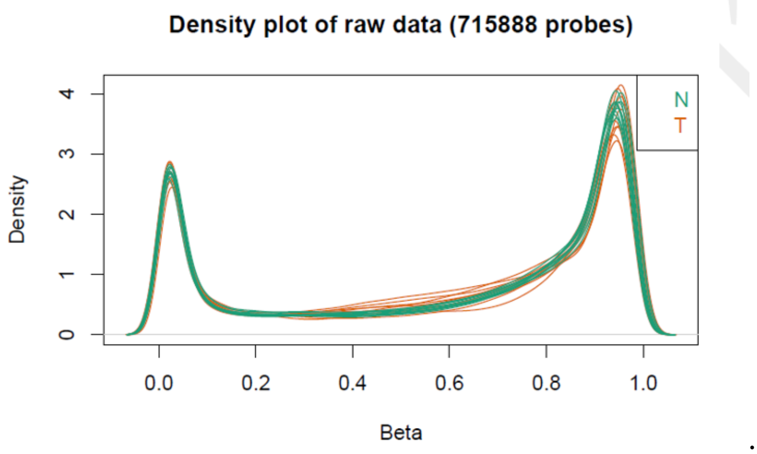

Figure S2. PCA analysis results of methylation ratio of each sample (Note: Color distinguishes sample groups; X and Y axes respectively correspond to the indicators that best reflect the accurate species composition of the sample, calculated by the software. The number represents patient's label number, and the following letter, N, represents patients' standard control while the letter T represents patients' thyroid carcinoma).

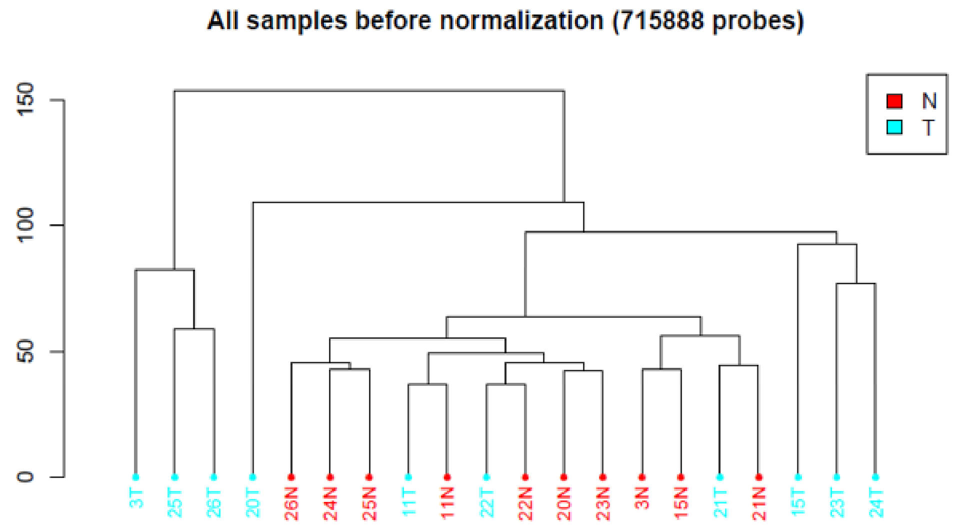

Figure S3. Hierarchical clustering results of methylation ratio of each sample. The number represents patient's label number, and the following letter N represents patients' standard control while the letter T represents patients' thyroid carcinoma.

Table S1. The number of qualified probes of the sample and the average intensity of all probes' red and green signals (Note: A maximum of 10 samples are displayed. "Sample Name" refers to the name of each sample; "Detected CpG (0.01)" refers to the number of qualified probes for the sample under the condition that the p-value threshold is 0.01, that is, the p-value of the retained probe in all samples is less than 0.01," Detected CpG (0.05)" refers to the number of qualified probes for the sample under the condition that the p-value threshold is 0.05, that is, the p-value of the retained probes in all samples is less than 0.05. "Signal Average GRN" refers to all the samples in each sample. The average intensity of the green signal of the probe, "Signal Average RED," refers to the average intensity of the red signal of all probes for each sample).

Sample Name	Detected CpG (0.01)	Detected CpG (0.05)	Signal Average GRN	Signal Average RED
11N	864526	864823	3490.015	3989.75
11T	863875	864324	4396.385	4715.449
15N	864141	864519	4732.551	5146.454
15T	864169	864459	5191.302	5789.714
20N	864345	864724	5465.038	6290.317
20T	864031	864742	5347.888	6133.555
21N	864438	864728	3007.487	3997.931
21T	864178	864669	3021.422	4119.732
22N	864300	864615	3287.029	4167.541
22T	863867	864440	3591.352	4357.978
